# The confinement induced resonance in spin-orbit coupled cold atoms with Raman coupling

**DOI:** 10.1038/srep04992

**Published:** 2014-05-27

**Authors:** Yi-Cai Zhang, Shu-Wei Song, Wu-Ming Liu

**Affiliations:** 1 Beijing National Laboratory for Condensed Matter Physics, Institute of Physics, Chinese Academy of Sciences, Beijing 100190, China

## Abstract

The confinement induced resonance provides an indispensable tool for the realization of the low-dimensional strongly interacting quantum system. Here, we investigate the confinement induced resonance in spin-orbit coupled cold atoms with Raman coupling. We find that the quasi-bound levels induced by the spin-orbit coupling and Raman coupling result in the Feshbach-type resonances. For sufficiently large Raman coupling, the bound states in one dimension exist only for sufficiently strong attractive interaction. Furthermore, the bound states in quasi-one dimension exist only for sufficient large ratio of the length scale of confinement to three dimensional s-wave scattering length. The Raman coupling substantially changes the confinement-induced resonance position. We give a proposal to realize confinement induced resonance through increasing Raman coupling strength in experiments.

The experimental realizations of synthetic gauge field and the spin-orbit coupling (SOC) in neutral cold atoms provide a new arena to explore the exotic effects in cold atomic physics. For example, the SOC could bring about nontrivial ground states in Bose-Einstein Condensate (BEC), such as vortex or vortex lattice states, plane wave phase, standing wave phase^1^,^2^,^3^,^4^,^5^,^6^,^7^,^8^,^9^,^10^. The prominent effect induced by the SOC in fermions is that the SOC could enhance the low energy density of states, which results in the formation of two-body bound states and enhancement of the pairing gap^11^,^12^. In polarized fermion gas, the spin-orbit coupling modifies the finite temperature phase diagram^13^. The exotic dynamic effect, e.g. *Zitterbewegung* oscillation, appears in the spin-orbit coupled cold atomic gas^14^,^15^. In the presence of SOC, the two-body scattering properties in three dimension have been investigated. It is shown that the SOC usually results in the mixed-partial-wave scattering^16^. For the low-energy scattering, the short range behaviors of wave function in three dimension can be modified by the SOC^17^,^18^,^19^. The two-body scattering properties in qusi-two-dimensional confinement with pure Rashba spin-orbit coupling are investigated^20^.

For the low-dimensional quantum gas, the two-body scattering properties can be affected greatly by the external confinement potential. For example, when the s-wave scattering length is comparable to the transverse confinement length (*a_s_*/*a*_⊥_ = 1/*C* with *C* = −*ζ*(1/2) ≈ 1.46), there exists a resonance, wherein the one-dimensional effective interaction constant diverges^21^,^22^. The similar scenario of the confinement induced resonance (CIR) also occurs in the quasi-two-dimensional case^23^,^24^. The confinement induced resonance has been observed through producing confinement induced molecules in quasi-one -dimensional Fermi gas^25^. It is also found experimentally that a single resonance splits into two resonances by introducing the anisotropic confinements^26^. The transversally anisotropic confinement alters the position of resonance by tuning the anisotropic ratio^27^,^28^. The confinement induced resonance which can be used to tune the interaction between atoms, provides a crucial ingredient to realize the strong interacting low-dimensional systems, such as Tonks-Girardeau gas^29^,^30^ and possible Tomonaga-Luttinger liquid^31^,^32^.

Some novel quantum states, for example, topological superfluidity, Majorana edge states or non-Abelian anyons could emerge in the low-dimension spin-orbit coupled quantum gas with Zeeman field^33^,^34^,^35^. In experiments, an effective Zeeman field in spin-orbit coupled atomic gas can be produced by two-photon Raman coupling^36^,^37^,^38^,^39^,^40^,^41^. The Raman coupling strength corresponds to the effective Zeeman field strength. The combination of the Raman coupling and SOC plays an essential role in the formation of the above novel quantum states. The effects induced by the Raman coupling and SOC are usually considered within BCS (Bardeen-Cooper-Schrieffer) mean-field framework^42^,^43^. It is known that the two-body interaction properties provide basis for understanding the many-body system. The studies on the effects of the Raman coupling on two-body problem may give some insight into exotic quantum states. In addition, the confinement induced resonance provides the indispensable tool for the realization of the low-dimensional strongly interacting quantum gas. Furthermore, how the Raman coupling and SOC affect confinement induced resonance is an inevitable question to clarify. In the present paper, we try to address the above questions by studying the two-body scattering problem in one dimension and the confinement induced resonance in the presence of the Raman coupling and SOC.

## Results

### The two-body scattering in the presence of spin-orbit coupling and Raman coupling

We consider the Hamiltonian of spin-orbit coupled cold atoms with Raman coupling 
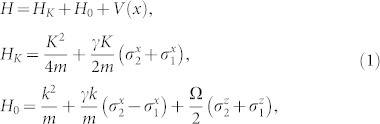
where the *H_K_* is the Hamiltonian in center of mass coordinate of two atoms, *H*_0_ is the free Hamiltonian in relative coordinate. *K* and *k* are the total momentum and relative momentum of two atoms along x direction, respectively. *σ^x^* and *σ^z^* are the spin Pauli matrix and *V*(*x*) is the interaction between two particles. *γ* denotes the SOC strength and Ω is the two-photon Raman coupling strength between two Zeeman sublevels in experiment. The SOC strength is determined by 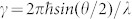, where *λ* is the Raman laser wave length, *m* is the mass of atom, *θ* is the angle between two Raman beams. The above Hamiltonian is realized experimentally in fermion atomic gas of ^40^K^38^. The above spin-orbit coupling is the mixture of equal Rashba and Dresselhaus type. Here, we choose the x axial direction as the direction of momentums. For simplicity, in the whole manuscript we denote *K* and *k* as the total and relative momentums, respectively, rather than *K_x_* and *k_x_*. The above Hamiltonian can be obtained from the Hamiltonian in Ref. 38 by applying a rotation in spin space (*σ^x^* → *σ^z^*, *σ^y^* → *σ^y^* and *σ^z^* → −*σ^x^*). We take the natural units *m* = 1, 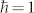 and *γ* = 1 in this section.

**Figure 1:**
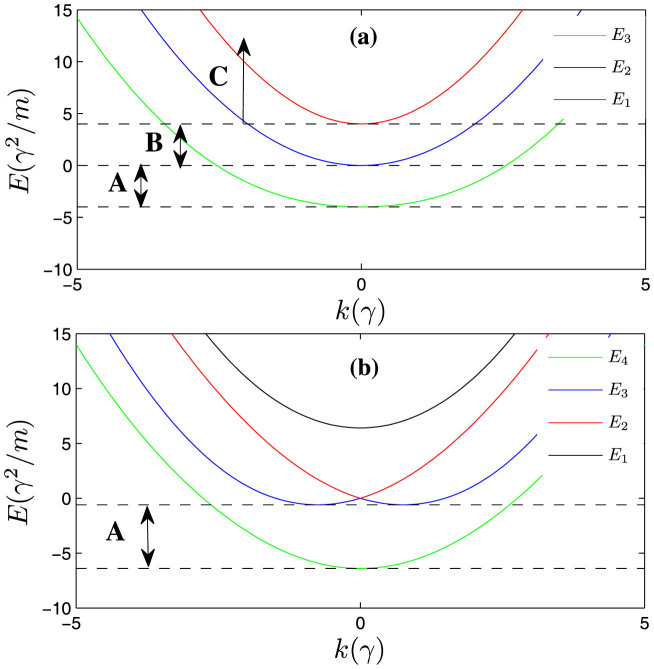
The energy spectrum of the relative motion (the Raman parameter Ω = 4*γ*^2^/*m*). (a): There are three energy branches (*K* = 0), the highest *E*_1_, the middle one *E*_2_ and the lowest branches *E*_3_. When the Raman coupling is weak (Ω ≤ 2*γ*), there exist two minimums on the lowest energy branch *E*_3_ (not shown in the Figure). When the Raman coupling is strong enough (Ω > 2*γ*), the lowest threshold is −Ω locating at the zero momentum of the lowest energy branch *E*_3_. The A, B and C label three different scattering energy intervals [−4, 0], [0, 4] and [4, ∞), respectively. The numbers of the scattering channels in different energy intervals are different. (b): Due to non-vanishing total momentum (*K* = 5*γ*), the spin-state |↑↓ + ↓↑〉 would couple with other spin states. So there would be four continuous energy branches.

From Eq. (1), we know that the motion of center of mass is coupled to the relative motion through spins. In the following, we focus on the subspace of Hamiltonian with *K* = 0 (the effects of the non-zero total momentum are also discussed). In the spin basis 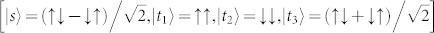, the Raman coupling and SOC are transformed into 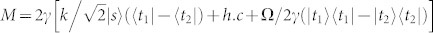. The interaction between cold atoms can be modeled by zero-range pseudo potential. Furthermore, we consider two identical spin-1/2 fermions. Hence, only the s-wave interaction in the singlet channel has contribution to two-body scattering. Therefore, the interaction matrix between two atoms takes the form as 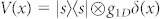, where *g*_1*D*_ is one-dimensional interaction constant. From the matrix *V* and *M*, we know that the spin channel |*t*_3_〉 is decoupled from other channels and not affected by the interaction. Thus, the spin channel |*t*_3_〉 is dropped in the following, and the Hamiltonian *H*_0_ is reduced to a 3 × 3 matrix. After diagnalizing *H*_0_, the eigenenerges are obtained as 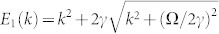, *E*_2_(*k*) = *k*^2^, 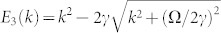 (see the panel (**a**) of Fig. 1), respectively. When the total momentum *K* ≠ 0, the spin channel |*t*_3_〉 will couple with other states. Then, the *H*_0_ is a 4 × 4 matrix and there are four continuous energy branches [see the panel (**b**) of Fig. 1].

The scattering problem can be solved through the Lipmann-Shwindger equation (for details see **Metholds**). The scattering with Raman coupling and SOC is intrinsically multi-channel scattering problem^44^,^45^. There exist different scattering thresholds for different energy branches. When the incident energy crosses the thresholds, some scattering channels are opened or closed. In certain scattering energy interval, there may exist several scattering channels scattering each other (see Fig. 1). The scattering amplitudes *f_m,n_* (reflection amplitudes) make up a matrix of rank 1, where the subscript *n* (*m*) denotes the specific incident (reflecting) channel with a specific energy 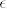. A single total amplitude *f* is obtained by diagnalizing the scattering matrix in every energy interval.

It is known that in the usual case without Raman coupling and SOC, the reflection coefficient approaches one (total reflection) as the incident energy approaches the scattering threshold of 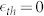. For a fixed incident energy 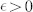, the reflection also approaches total reflection as the interaction *g*_1*D*_ approaches infinity. For attractive interaction *g*_1*D*_ < 0, there always exists a bound state below the scattering threshold.

**Figure 2:**
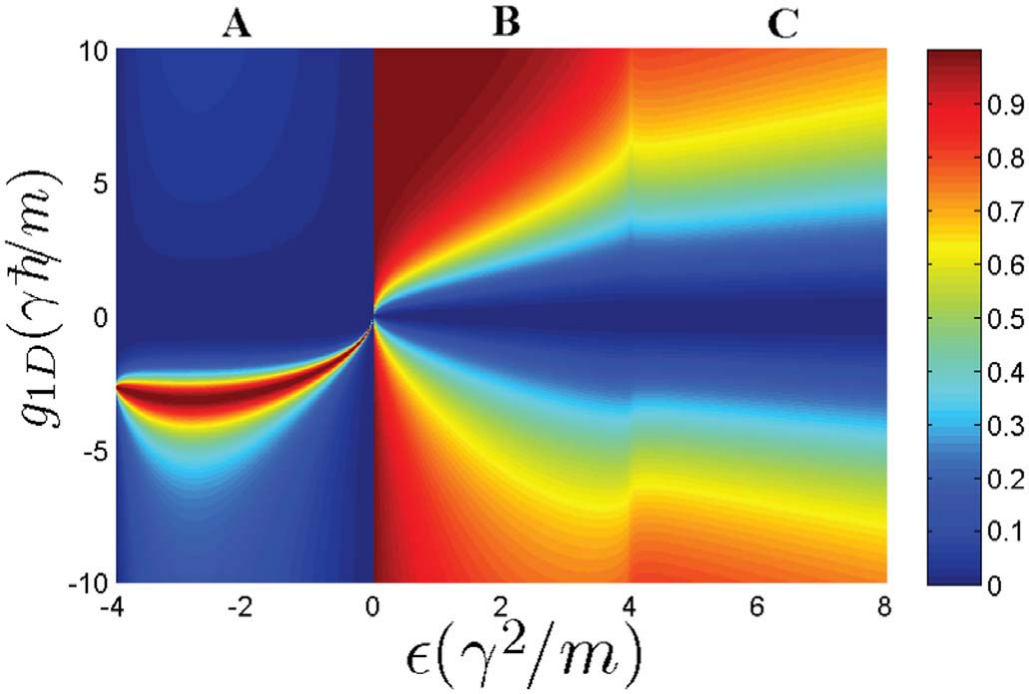
The reflection coefficient |*f*|^2^ as a function of the interaction *g*_1*D*_ and the scattering energy 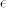 when Ω = 4*γ*^2^/*m* (*K* = 0). *f* is the scattering (reflection) amplitude in one dimension. The corresponding three scattering energy intervals in panel (a) of Fig. 1 are also labeled here.

**Figure 3:**
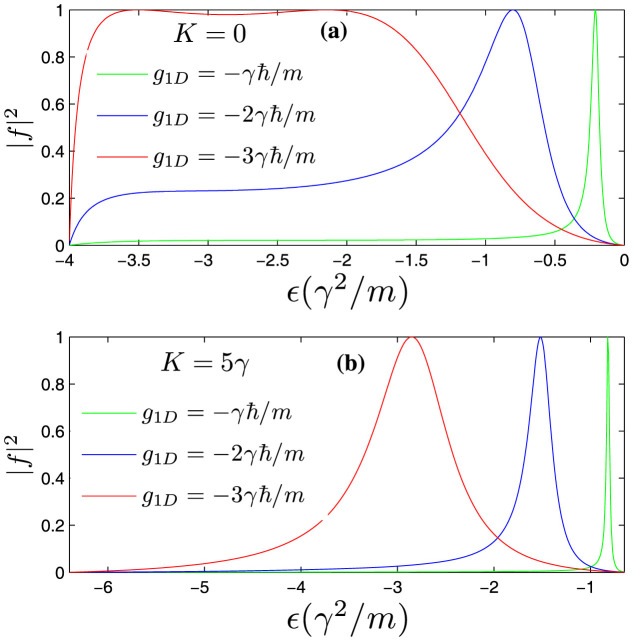
The reflection coefficients in the energy interval A of Fig. 1. (a): the refection coefficient corresponding to *K* = 0. (b): the refection coefficient corresponding to *K* = 5*γ*. The Feshbach resonance peaks correspond to the quasi-bound states embedded between the continuous energy branches. The resonance position and width vary with the increase of the interaction strength. We can see that when the incident energy 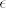 approaches the threshold of the middle branch *E*_2_, the reflection vanishes. When the incident energy 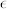 approaches the lowest threshold the reflections are not total reflections.

In Figs. 2 and 3, we calculate the reflection coefficient (|*f*|^2^) from the obtained scattering amplitude *f*. We can see that, compared with the usual case, the Raman coupling and SOC cause fundamental changes in the behaviors of the scattering amplitude at low energy. **First of all**, there exist scattering resonances in the parameter space because there exist quasi-bound states between the energy branches. The interaction matrix *V* can be rewritten in terms of the eigen-basis of *H*_0_. In addition to the interaction in the respective eigen-basis channel (diagonal part), there are also non-diagonal part coupling to different eigen-basis. The upper energy branches could support bound states near the incident energy. In addition, the bound states are coupled to the scattering states due to the non-diagonal interaction term. Hence, it results in a Feshbach-type resonance scattering^46^,^47^,^48^. **Secondly**, when the collision energy approaches the lowest threshold, the reflection coefficient does not approach 1. So the incident wave could not be totally reflected in the presence of SOC and Raman coupling. **Thirdly**, the reflection may vanish under certain conditions. As shown in Fig. 2 and panel (**a**) of Fig. 3, as the incident energy approaches the threshold of 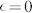 from below, the scattering amplitude becomes zero no matter how large the interaction is. This is because when the incident energy approaches the threshold of zero energy from below (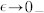), the middle energy branch *E*_2_ contributes a infinitely large real number 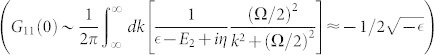 to the denominator of the scattering amplitude (see Eq. (4) in **Metholds**). **Next**, as the interaction *g*_1*D*_ approaches infinity, contrary to the usual case, the reflection needs not mean a total reflection. Due to the coupling between singlet and triplet channels caused by the SOC, the incident wave can tunnel through other channels, even the interaction strength is very strong in singlet channel. A similar scenario also appears in impurity scattering problems of multi-component coupled system^49^.

**Finally**, the large Raman coupling changes the conditions of the existence of bound states. When the Raman coupling satisfies 0 < Ω ≤ 2*γ*, there always exists a bound state below the lowest threshold for attractive interaction *g*_1*D*_ < 0 as the usual case. However, if Ω > 2*γ* > 0, there exists a bound state only when the attractive interaction is strong enough. This is because when the energy approaches the lowest threshold (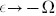), all the energy branches contribute a finite part 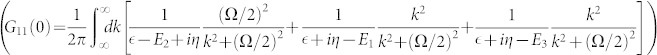 to the denominator of the scattering amplitude (see Eq. (4) in **Metholds**), rather than an infinitely large number as that in usual case without Raman coupling and SOC. The existence of the bound states is directly related to the density of states near the lowest scattering threshold. It is known that, in the usual case, the density of states in one dimension near the scattering threshold (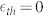) behaves like 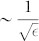. There always exists a bound state bellow the scattering threshold 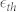 as long as the interacting (*g*_1*D*_ < 0) is attractive (no matter how weak it is). When the Raman coupling is weak (Ω ≤ 2*γ*), the lowest energy branch has two minimums occurring at non-zero momentums. The density of states near the minimums also looks like 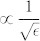 (the 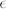 is measured with respect to the minimum of the continuous spectrum).

Then the conditions of existence of bound states are the same as the usual case. However, if the Raman coupling is strong enough (Ω > 2*γ*), the lowest energy branch has only one minimum locating at the zero momentum (see the Fig. 1). Intuitively thinking, the density of states should be proportional to 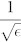. However, at the zero momentum, we can see that the spin-orbit coupling does not play role at all in the energy spectrum (see Eq. (1)). The lowest point should correspond to the spin-triplet state, rather than the singlet state. Furthermore, we consider the interaction occurring only in the singlet channel. So the effective density of states near the lowest threshold which could contribute to the formation of bound state is very low. For example, there exists a suppression factor 
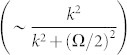
 in the denominator of the scattering amplitude (see Eq. (4) in **Metholds**). The effective density of states near the lowest scattering threshold behaves like 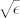 approaching to zero as 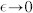 (the 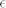 is measured with respect to the lowest scattering threshold), rather than like 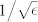 blowing up. So, in this case, there exist bound states only when the interaction is strong enough. In the following, we will see that the modifications of effective density of states near the lowest threshold also have significant impacts on the existence of the bound states of quasi-one-dimensional system.

**Figure 4:**
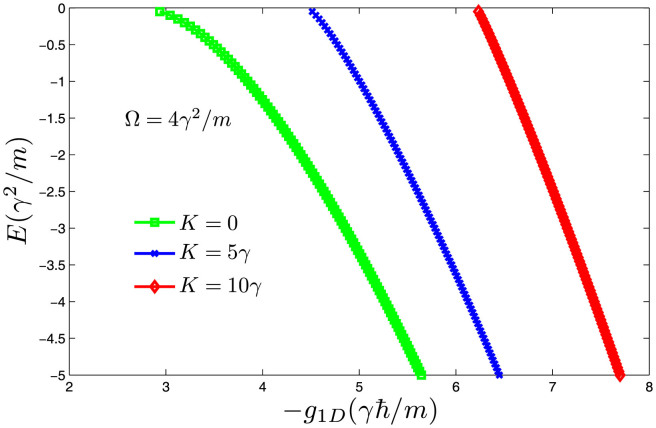
The energy of bound states for attractive interaction (*g*_1*D*_ < 0) in one dimension From the Fig. 4, we can see the energy of bound states is getting lower and lower with increase the interaction magnitude |*g*_1*D*_|. The critical interaction magnitude is large for large total momentum *K*. So the effects of *K* are very similar to that of increasing Raman coupling.

The modifications of scattering properties are robust even for non-zero total momentum *K* ≠ 0 (see the panel (**b**) of Fig. 3). However, the position and width of Feshbach Resonance are usually modified by the non-zero total momentum. Fig. 4 shows the energy of bound states (with respect to the lowest scattering threshold) for attractive interaction (*g*_1*D*_ < 0) in the pure one-dimensional system. We can see that, with increase of total momentum *K*, the critical interaction magnitude |*g*_1*D*_| where the bound states begin to appear near the lowest threshold becomes larger and larger. From the above discussion, we know that for stronger Raman coupling Ω, the required interaction strength |*g*_1*D*_| should be stronger in order to form a bound state. So the effects of the *K* ≠ 0 is similar to that of increasing Raman coupling. In the next section, we will see the conclusion that the increase of total momentum *K* amounts to increasing the Raman coupling Ω is also valid for the quasi-one-dimensional system.

### The confinement induced resonance

The one-dimensional effective interaction constant is derived through investigating two-body problem of three dimension with confinement. The Raman coupling and SOC may change the condition of confinement induced resonance. After separating the motion of center of mass, the Hamiltonian with confinement can be written as 
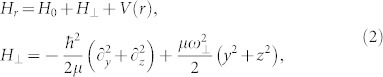
where *H*_0_ is the free Hamiltonian of relative coordinate along x direction as above section, *H*_⊥_ is transverse confinement, 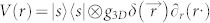 is the three dimensional s-wave psuedo-potential interaction between atoms^50^,^51^. 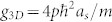 and *ω*_⊥_ are the three dimensional interaction constant and frequency of confinement trap, respectively. *a_s_* and *μ* = *m*/2 are the s-wave scattering length and the reduced mass of two atoms, respectively. In this section, we take natural units as *m* = 1, 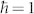 and *ω*_⊥_ = 1.

The one-dimensional effective interaction constant *g*_1*D*_ can be obtained in terms of the three dimensional s-wave scattering length *a_s_* (see **Methods**), 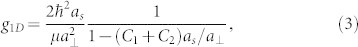
with 
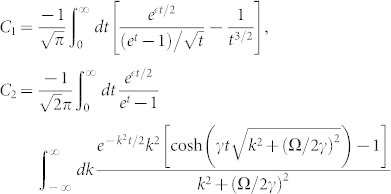
The above equation gives the connection between the one-dimensional effective interaction constant *g*_1*D*_ and the three dimensional s-wave scattering length. When the SOC vanishes (*γ* = 0), *C*_2_ becomes zero. Furthermore, if the scattering energy 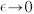, the constant *C*_1_ = *C* = −*ζ*(1/2) ≈ 1.46, the resonance condition is reduced to Olshanii's result^21^.

**Figure 5:**
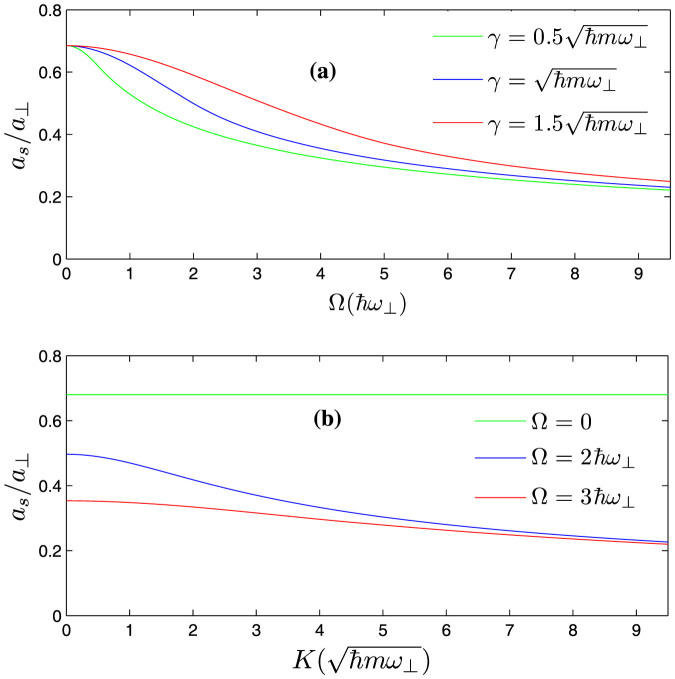
The resonance position *a_s_*/*a***_⊥_** as a function of the Raman parameter Ω and total momentum *K* (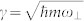). (a): as shown in panel (a) of Fig. 5, when the Raman coupling is absent (Ω = 0) and the incident energy approaches the lowest threshold (the minimum of *E*_3_), the resonance condition is also exactly the same as the case without SOC (*a_s_*/*a*_⊥_ = 1/(*C*_1_ + *C*_2_) = 1/*C* ≈ 0.68). It is related to the fact that the constant gauge potential can be gauged away by applying a gauge transformation when the Raman coupling is absent. However, in the presence of non-zero Raman coupling, the resonance condition at the lowest threshold could never recover the usual case. (b): the resonance position as a function of total momentum *K*. For non-vanishing Raman coupling (Ω ≠ 0), the resonance position *a_s_*/*a*_⊥_ is getting smaller smaller with the increase of the total momentum *K*.

From panel (**a**) of Fig. 5, we can find that, with the increase of the Raman coupling, the resonance position *a_s_*/*a*_⊥_ is getting smaller. In addition, the resonance position inclines to be independent of SOC strength for sufficiently large Raman coupling (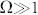). In fact, the resonance position 
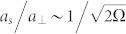
 can be arbitrarily small by increasing the Raman parameter Ω for fixed SOC parameter (*γ* ~ 1). It can be shown that, for a fixed spin-orbit coupling (*γ* = 1), 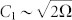 diverges and *C*_2_ is bounded as Ω → ∞. When the condition of confinement induced resonance is satisfied, from Eq.(3), the resonance position 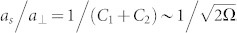. It means that, compared with the usual case, it is much easier to fulfill the conditions of confinement induced resonance in the presence of Raman coupling and SOC. For sufficiently large Raman coupling, the experimental observation of confinement induced resonance needs not resort to the usual magnetic Feshbach resonance techniques.

**Figure 6:**
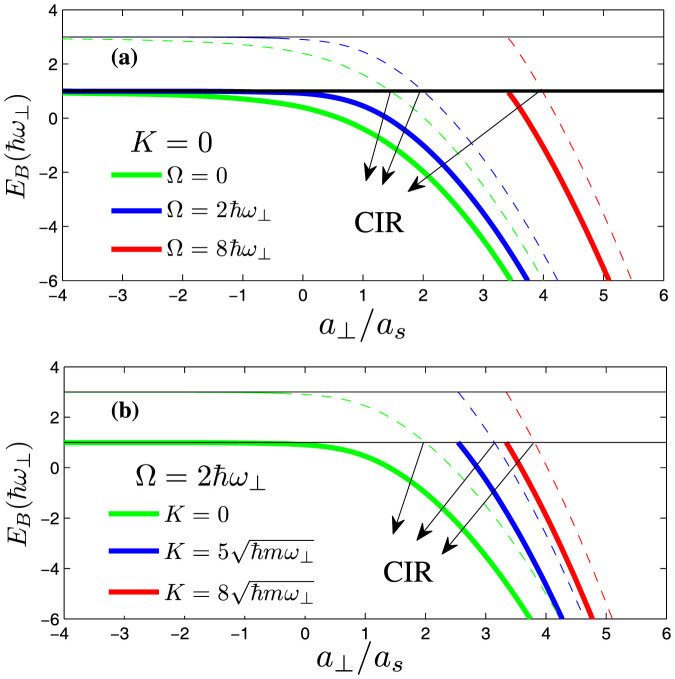
The bound state energies in the closed channels and the full Hamiltonian, respectively (with SOC strength 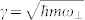). (a): The bound state energies are measured with respect to the lowest thresholds. The thin lines denote the bound state energies supported by the closed channels, and the thick lines are the bound state energies in the full Hamiltonian. With the increase of Raman coupling Ω, the resonance position *a_s_*/*a*_⊥_ (the reciprocal of *a*_⊥_/*a_s_*) is getting smaller and smaller, which is consistent with that in panel (a) of Fig. 5. (b): the energy of bound states for various total momentums *K*. We can see that with the increase of the total momentum *K*, the modifications of existing conditions of bound states are very similar to that of increasing the Raman coupling. **Note** here the green line in panel (b) corresponds to the blue line in panel (a).

For fixed SOC strength (*γ* = 1), we show in Fig. 6 the bound state energies supported by the “closed” channels (the transversely excited modes) and the full Hamiltonian, respectively^22^,^27^. The confinement induced resonance can also be viewed as a Feshbach resonance as that in the usual case without Raman coupling and SOC. The resonance condition is satisfied when the energy of the bound states in closed channel coincides with the scattering threshold of the ground transverse modes. In the meantime, the difference between the bound state energies in the closed channels and the full Hamiltonian is 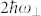.

However, based on the same reasons as that in the pure one-dimensional case, the large Raman coupling also changes the existing condition of the bound states in the quasi-one dimension. When the Raman coupling satisfies Ω ≤ 2*γ*, there always exist the bound states irrespective of the sign of the s-wave scattering length *a_s_*, which is consistent with the usual case^25^ (see the green and blue lines in the panel (**a**) of Fig. 6). However, if Ω > 2*γ*, the bound states exist only for sufficiently large *a*_⊥_/*a_s_* (see red lines). As stated before, the reason is that the spin-orbit coupling and the Raman coupling change greatly the effective density of states near the lowest scattering threshold of energy spectrum. The spin-orbit coupling term 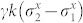 does not play significant roles near zero momentum. So the states corresponding to the lowest and highest spectrum belong to spin-triplets, while the state in the middle energy branch is the spin-singlet. In addition, we consider the interactions occurring at the spin-singlet channel. So the bound states can be roughly viewed as the spin-singlet bound states which are mainly supported by the middle energy spectrum. Furthermore, the bound states exist below the lowest threshold (the threshold of triplet energy spectrum). So, the binding energy (relative to the middle energy branch) of bound states has to be larger than the differences between the thresholds of the middle and the lowest energy branches. In other words, the large Raman coupling Ω pushes the energy of bound state low by amount of Ω at least. So, for strong Raman coupling, there exist bound states only when the interaction is strong enough. Then the bound states belong to the deeply binding states of the spin-singlet channel. In consequence, the position of confinement induced resonance is also modified accordingly.

The panel (**b**) of Fig. 5 shows the dependence of the resonance position on the variation of the total momentum *K*. When the Raman coupling Ω = 0, the spin-orbit coupling terms can be removed by a gauge transformation. So the resonance position is not affected by the total momentum *K* (see the green line in the panel (**b**)). When the Raman coupling Ω ≠ 0, the resonance position *a_s_*/*a*_⊥_ becomes smaller and smaller with the increase of *K*. The panel (**b**) of Fig. 6 shows the energy of bound states for *K* ≠ 0 in the quasi-one-dimensional system. Similar to the effects of increasing the Raman coupling Ω, the strong total momentum *K* also modifies the existing condition of bound states in quasi-one dimension. So the increase of total momentum *K* amounts to the increase of Raman coupling Ω if the Raman coupling Ω ≠ 0.

Here we see what roles the confinement and the SOC (Raman coupling) play, respectively. The confinement freezes the transverse degree of atomic motion, and produces a quasi-one-dimensional system. Due to the enhancement of density of states, there is always two-body bound state in the quasi-one-dimensional system no matter how weak the interaction is, as long as the interaction is attractive. After introducing the SOC and Raman coupling, the couplings could reduce, or even change fundamentally the effective density of states near the lowest threshold. The existence condition of bound states is also modified accordingly. The above two competing factors determine the formations of bound states in the confined spin-orbit coupled system.

## Discussion

The Raman coupling and SOC have been realized experimentally in^40^K atomic gas^38^. Two magnetic sublevels |↑〉 = |9/2, 9/2〉 and |↓〉 = |9/2, 7/2〉 are chosen as two spin 1/2 states. One can choose the experimental parameters 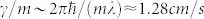, *ω*_⊥_ ~ 2*π* × 17 kHz with *a*_⊥_ ~ 172 *nm*. The s-wave background scattering length *a_s_* ~ 170*a*_0_ ~ 9 *nm*. Under the above conditions, the SOC strength 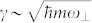, the ratio *a_s_*/*a*_⊥_ = 0.052. To satisfy the condition of confinement induced resonance, one can increase Raman coupling strength (Ω ~ *h* × 3 MHz) by tuning the intensity of Raman beams. The binding energies of bound states near confinement induced resonance (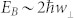) can be measured by using radio-frequency (rf) spectroscopy^52^. It is expected that there are two peaks in the radio-frequency photodissociation spectra. One peak locates at the atomic transition frequency (*ν*_0_) of an occupied state to another initially unoccupied state (e.g. |9/2, 7/2〉 → |9/2, 5/2〉). The other peak at a non-zero detuning (*δ* = *ν_rf_* − *ν*_0_ ~ 34 kHz) from the atomic transition corresponds to the dissociation of quasi-one-dimensional bound states^25^.

In summary, we investigate the effects of SOC and the Raman coupling on the confinement induced resonance. The Raman coupling and spin-orbit coupling fundamentally change the interacting properties of atoms. We propose to realize the confinement induced resonance by increasing Raman coupling strength. Different from the usual way, such as utilizing Feshbach resonances to produce a large scattering length, our work gives a new way to realize the strongly interacting quasi-one-dimensional atomic gas with Raman coupling and spin-orbit coupling. Due to the exotic effects induced by Raman coupling and spin-orbit coupling, a lot of interesting many-body physical phenomena, e.g., the crossover of BCS-BEC superfluidity^53^,^54^, inhomogeneous Fulde-Ferrell-Larkin-Ovhinnikov (FFLO) state^55^, fermion pair breaking in the presence of external magnetic field^56^, need to be revised in the strong interacting quasi-one dimension atomic gas.

## Methods

In this work, we investigate the two-body scattering problem in one-dimensional and the three dimensional cold atomic systems with confinement, respectively. Through comparing the scattering state in the one dimension with that in three dimension, one can get the effective one-dimensional constant (*g*_1*D*_) in terms of the three dimensional s-wave scattering length (*a_s_*).

### 1 Two-body scattering in one dimension

In this section, we give the detailed calculation of the scattering amplitude. We consider the one-dimensional Hamiltonian of two atoms in the presence of the Raman coupling and the spin-orbit coupling 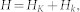


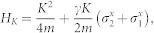


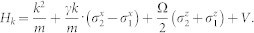
When the total momentum *K* = 0, the Hamiltonian of center of mass (*H_K_*) is zero. In the spin basis 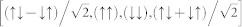, the Schrödinger equation in the relative coordinate is 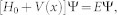


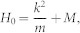
with 
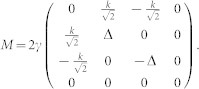
We introduce 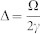 in the above equation.

The interaction between atoms can be modeled by zero-range pseudo potential. Therefore the interaction matrix between two atoms takes the form as 
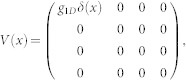
where *g*_1*D*_ is the one-dimensional interaction constant. From the interaction matrix *V* and matrix *M*, one can get that the spin channel 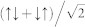 is decoupled from other channels in the case of *K* = 0. So we will drop it in the following. The Hamiltonian is reduced a 3 × 3 matrix. After diagnalizing the *H*_0_, the eigenstates (in coordinate space) and eigenenerges are obtained 
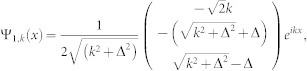


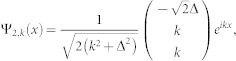


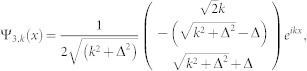
corresponding to eigensnergies 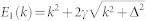, *E*_2_(*k*) = *k*^2^, 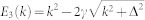, respectively (in the natural units of *m* = 1, 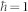 and *γ* = 1). The energy spectrum is shown in panel (**a**) of Fig. 1.

The scattering problem can be solved through the Lipmann-Shwindger equation 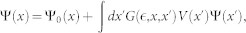
where 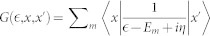 is the free Green's function. In general, the Green's function is a 3 × 3 matrix. In the case of zero range interaction the Lipmann-Shwindger equation takes the following form 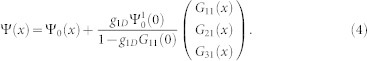
where 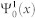 is the first component of incident state Ψ_0_(*x*) and 
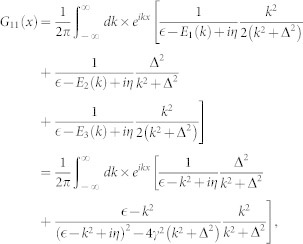


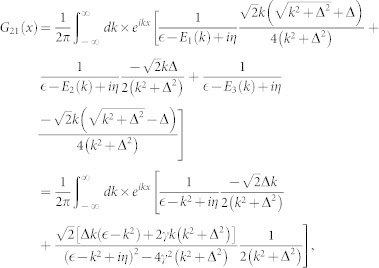


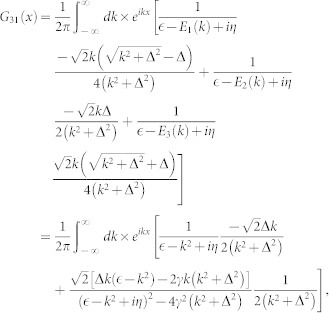
with *η* → 0_+_. The above integrals can be obtained by using the residue theorem 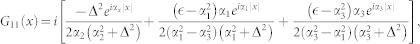


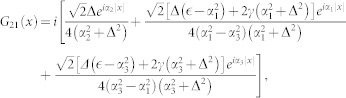


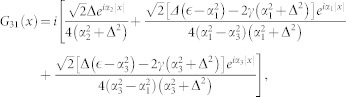
with 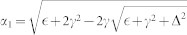, 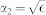 and 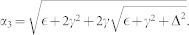 For *x* > 0, the above equations is expressed in another form 
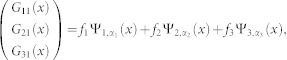
where 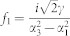, 
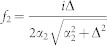
, 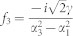. For a given incident energy, there is possibility that there exist several states scattering each other (see Fig. 1). From the Eq. (4), we can explicitly obtain the scattering amplitude for a given incident state. The scattering amplitude makes up to a scattering matrix of rank 1, it can be reduced to one single total amplitude *f* by diagnalizing the scattering matrix. According to the number of the scattering states, there are several distinct cases.

#### The incident energy is above the highest scattering threshold 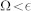 [within energy interval C (see Fig. 1)]

When incident energy satisfies 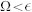, there will be three eigen-states scattering each other forwardly (all *α*_1_, *α*_2_ and *α*_3_ are real number), which locate at the right semi-axis of relative momentum (*k* > 0) (see Fig. 1). The resulting total scattering amplitude is 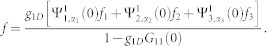


#### The incident energy is above the zero-energy, but below the highest threshold Ω (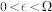 within energy interval B)

When incident energy lies in 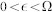, the scattering state on the highest branch is closed (*α*_1_ becomes imaginary number). The remainder two eigen-states scattered each other forwardly. The scattering amplitude is 
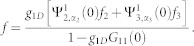


#### The scattering energy satisfies 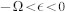 (within energy interval A)

When the scattering energy *ε* lies in the regime 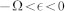, there is only one scattering state on the lowest energy branch (both *α*_1_ and *α*_2_ become imaginary number). The scattering amplitude is 
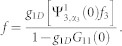


### 2 The confinement induced resonance

The scattering problem in three dimension with confinement can be solved as follow. We assume the confinement is strong enough that only the transverse harmonic ground state is occupied. The incident energy with respect to the lowest threshold and the ground state energy of transverse harmonic oscillator should be lower than the transverse exited state energy. The lowest excited state which can be coupled to ground states by the s-wave interaction is *ϕ*_1_(*y*)*ϕ*_1_(*z*) with energy 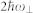
22,57. Then, the incident energy of scattering states should 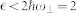 (in natural units of *m* = 1, 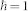 and *ω*_⊥_ = 1). Using the Lipmann-Shwindger equation, three dimension scattering wave function can be obtained 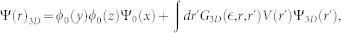
where 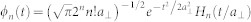, 
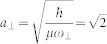
 is wave function and the length scale of transverse Harmonic oscillator. Ψ_0_(*x*) is one dimensional incident wave function along x direction as above section. The interaction *V* is the pseudo-potential 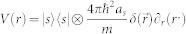. Substituting the interaction *V*, the wave function in three dimension becomes 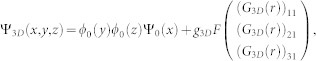
where 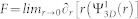, 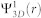 is first component of three dimensional wave function. Focusing on *y* = *z* = 0 and the resulting qusi-one dimensional wave function is 
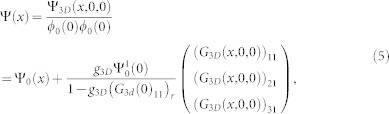
where (*G*_3*D*_(0)_11_)*_r_* = *lim_r_*_→0_*∂_r_*[*r*(*G*_3*D*_(*r*)_11_)] is the regular part of the Green's function matrix element *G*_11_ at origin. It is known that when *r* → 0, the three dimensional Green's function in confined system also diverges as that in homogeneous space (*G*_3*D*_(*r*)_11_)*_r_*_→0_ ∝ − 1/4*πr*^58^. In order to obtain the regular part, we need to subtract the singular part (−1/4*πr*) from *G*_3*D*_(*r*)_11_ near the origin. For the qusi-one dimensional scattering problem, we only need to know the long-ranged asymptotic behavior of three dimensional Green's function and the regular part of *G*_3*D*_(*x*, *y* = 0, *z* = 0)_11_ near *x* = 0. The Green's function can be decomposed as two terms: 
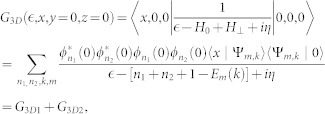
where 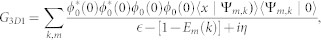
and 
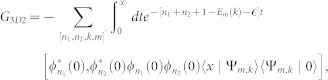
here |Ψ*_m_*_,*k*_〉 are the eigenstates of *H*_0_ which is same as that in the above section. The summation 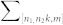 excludes the term [*n*_1_ = 0, *n*_2_ = 0, *k*, *m*], the identity 
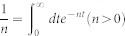
 has been used in the above Equation. The first part *G*_3*D*1_ is related to the scattering channel and long ranged, while the second part *G*_3*D*2_ is short ranged corresponding to influences of virtual transition of other closed channels. Redefining the reference point of energy 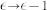, it is easy to see that the first term *G*_3*D*1_ is just the one dimensional Green's function *G*(*x*) up to a factor |*ϕ*_0_(0)|^4^ = 1/2*π*. In the following, we will see the short-ranged part of matrix element (*G*_3*D*2_(*x*))_11_ is related to confinement induced resonance (CIR).

Using 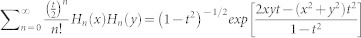, completing the summation over the index [*n*_1_, *n*_2_], the second part of the matrix element (*G*_3*D*2_(*x*))_11_ takes the form 
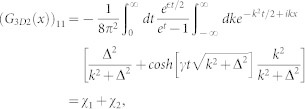
where 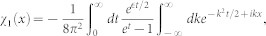
and 
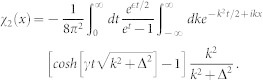
It can be shown that the *χ*_2_(*x*) is not singular as *x* → 0, while the *χ*_1_(*x*) does diverge *χ*_1_(*x*) ∝ −1/4*πx*. Substracting the diverging part, 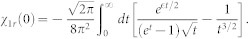
So the regular part of Green's function (*G*_3*D*_(0)_11_)*_r_* = *G*_11_(0)|*ϕ*(0)|^4^ + *χ*_1*r*_(0) + *χ*_2_(0). For 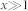, the Green's function 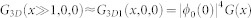, *G*(*x*) is the Green's function in pure one domension. Comparing Eq. (4) with (5), we can get the one-dimensional effective interaction constant in term of the three dimensional s-wave interaction constant 
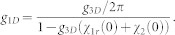
Introducing 
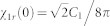
, 
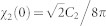
 and using 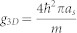, 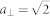, *m* = 2*μ* and 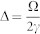, the above equation is reduced to Eq. (3) in the main text. 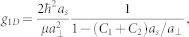
with 
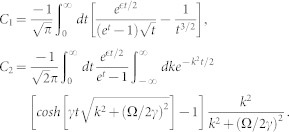


## Author Contributions

Y.C.Z. performed calculations. Y.C.Z., S.S.W., W.M.L. analyzed numerical results. Y.C.Z., S.S.W., W.M.L. contributed in completing the paper.
